# Differential Proteomic Analysis of Platelets Suggested Possible Signal Cascades Network in Platelets Treated with Salvianolic Acid B

**DOI:** 10.1371/journal.pone.0014692

**Published:** 2011-02-17

**Authors:** Chao Ma, Yan Yao, Qing-Xi Yue, Xin-Wen Zhou, Peng-Yuan Yang, Wan-Ying Wu, Shu-Hong Guan, Bao-Hong Jiang, Min Yang, Xuan Liu, De-An Guo

**Affiliations:** 1 Shanghai Research Center for Modernization of Traditional Chinese Medicine, Shanghai Institute of Materia Medica, Chinese Academy of Sciences, Shanghai, People's Republic of China; 2 Institutes of Biomedical Sciences, Fudan University, Shanghai, People's Republic of China; Universidad Peruana Cayetano Heredia, Peru

## Abstract

**Background:**

Salvianolic acid B (SB) is an active component isolated from Danshen, a traditional Chinese medicine widely used for the treatment of cardiovascular disorders. Previous study suggested that SB might inhibit adhesion as well as aggregation of platelets by a mechanism involving the integrin α2β1. But, the signal cascades in platelets after SB binding are still not clear.

**Methodology/Principal Findings:**

In the present study, a differential proteomic analysis (two-dimensional electrophoresis) was conducted to check the protein expression profiles of rat platelets with or without treatment of SB. Proteins altered in level after SB exposure were identified by MALDI-TOF MS/MS. Treatment of SB caused regulation of 20 proteins such as heat shock-related 70 kDa protein 2 (hsp70), LIM domain protein CLP-36, copine I, peroxiredoxin-2, coronin-1 B and cytoplasmic dynein intermediate chain 2C. The regulation of SB on protein levels was confirmed by Western blotting. The signal cascades network induced by SB after its binding with integrin α2β1 was predicted. To certify the predicted network, binding affinity of SB to integrin α2β1 was checked *in vitro* and *ex vivo* in platelets. Furthermore, the effects of SB on protein levels of hsp70, coronin-1B and intracellular levels of Ca(2+) and reactive oxygen species (ROS) were checked with or without pre-treatment of platelets using antibody against integrin α2β1. Electron microscopy study confirmed that SB affected cytoskeleton structure of platelets.

**Conclusions/Significance:**

Integrin α2β1 might be one of the direct target proteins of SB in platelets. The signal cascades network of SB after binding with integrin α2β1 might include regulation of intracellular Ca(2+) level, cytoskeleton-related proteins such as coronin-1B and cytoskeleton structure of platelets.

## Introduction

Salvianolic acid B (SB) is one of the main components of Danshen (root of *Salvia miltiorrhiza*), a popularly used traditional Chinese medicine for patients with coronary artery disease in both China and other countries including the U.S. [1]-[4]. The mechanism of Danshen is still not clear but its inhibitive effect on platelet adhesion and aggregation might be one of the important bases for its cardiovascular effects. Both crude extract of Danshen [5]-[7] and purified compounds such as SB [8], [9] were reported to exhibit inhibitory effects on platelet aggregation and adhesion. Our previous study also showed that salvianolic acids isolated from *Salvia miltiorrhiza* could inhibit ADP-induced platelet aggregation of rat platelets both *in vitro* and *ex vivo* [10]. Furthermore, our cooperation study result suggested that SB might inhibit platelet adhesion by a mechanism involving the integrin α2β1 (collagen receptor α2β1) [9].

To further clarify the signal cascades in SB-treated platelets, a proteomic approach was used in the present study to identify regulated proteins after exposure of intact platelets to SB. The result of proteomic analysis was confirmed by using Western blotting assay. And, comparing with our previous proteomic study using a mixture of salvianolic acids [11], the results suggested that the most important target-related proteins of SB might be heat shock-related 70 kDa protein 2, LIM domain protein CLP-36, copine I, peroxiredoxin-2, coronin-1 B and cytoplasmic dynein intermediate chain 2C. The possible signal cascades net work in platelets after binding of SB to integrin α2β1 was predicted. To certify the predicted network, the binding affinity of SB to integrin α2β1 was checked both *in vitro* and *ex vivo* in platelets. Furthermore, the effects of SB on platelets such as changing protein levels and changing intracellular Ca (2+) level, ROS level as well as cell size were observed with or without pre-treatment of platelets using an antibody against integrin α2β1.

## Results

### Effect of SB on platelet adhesion and aggregation

**Figure 1:**
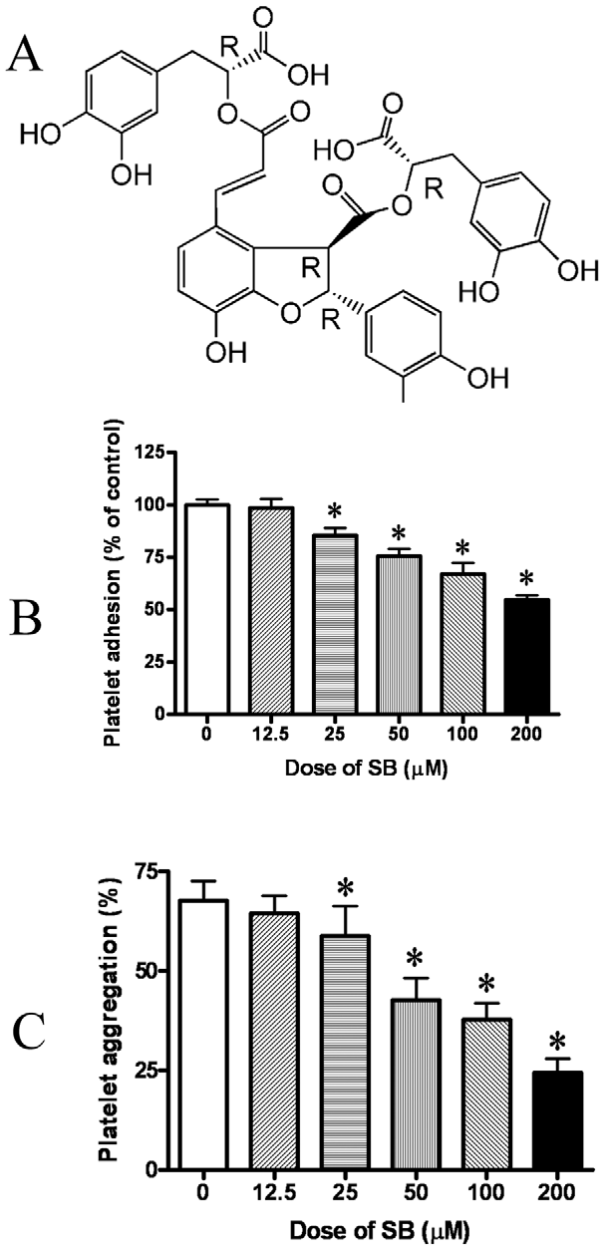
The inhibitive effect of SB on platelet adhesion and platelet aggregation. A, chemical structure of SB. B, SB dose-dependently inhibited adhesion of rat washed platelets. C, SB dose-dependently inhibited aggregation of rat platelets induced by collagen. Data represent mean ± S.D. (n = 6). *Significant difference from the control group at P<0.05.

As shown in B of Fig. 1, SB dose-dependently inhibited the adhesion of platelets on collagen. And, as shown in C of Figure 1, SB also could dose-dependently inhibit the aggregation of platelets induced by collagen.

### Protein expression profiles of platelets and identification of the differentially expressed proteins

**Figure 2:**
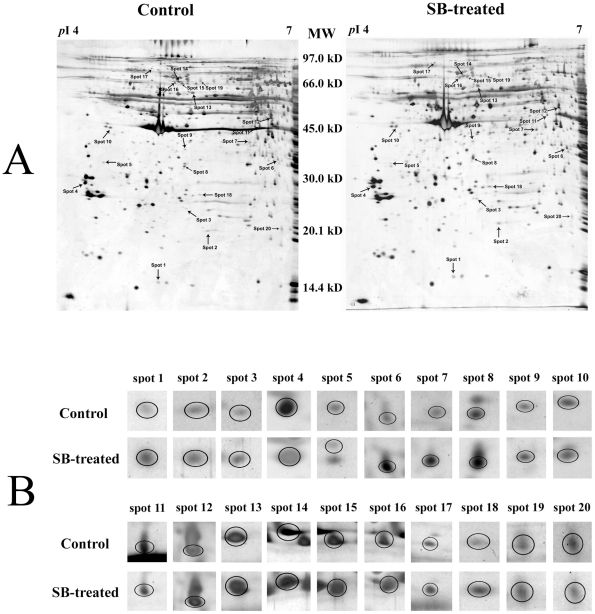
The proteome maps of control and SB-treated platelets. A, Representative two-dimensional electrophoresis gel pair images. Differentially expressed spots were shown by the arrows. B, The expanded region of differentially expressed protein spots in A. The proteins within the circles were the differentially expressed proteins.

**Figure 3:**
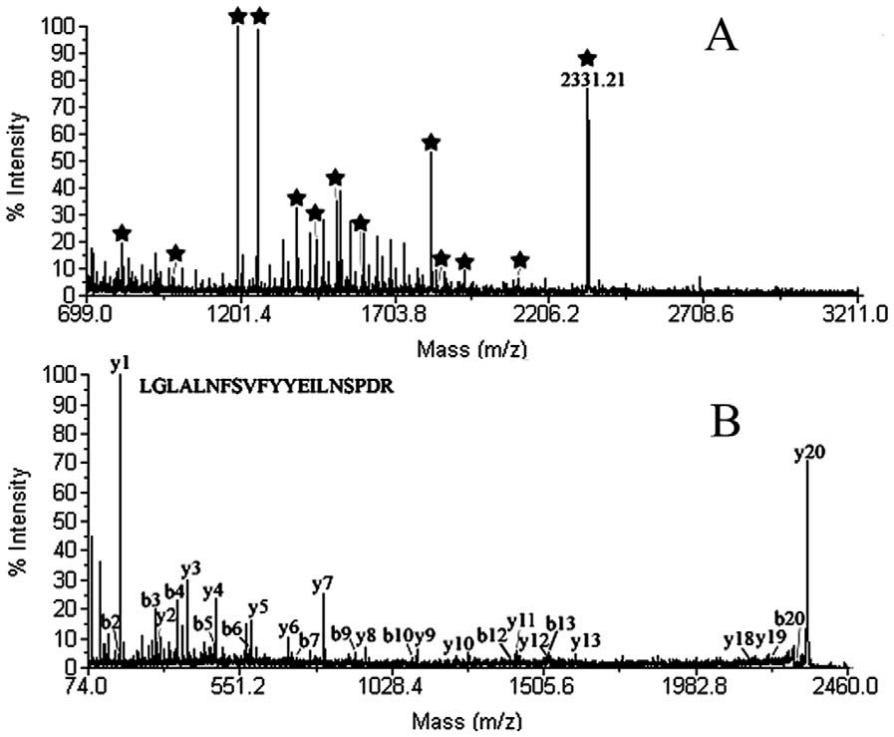
The result of the MALDI-TOF MS/MS analysis of the protein spot 4. It was identified to be rat 14-3-3 epsilon (NCBI accession number 13928824) by protein database search. A, Peptide mass fingerprint of the tryptic digest of spot 4. Peptide signals identified were marked with asterisks. B, MS/MS profile of the peptide with a mass of 2331.21 Da. y-ions resulting from fragmentation of the peptides and amino acids they represent are indicated.

Representative two-dimensional electrophoresis gel images for control, SB-treated platelets were shown in A of Fig. 2. Fifteen up-regulated protein spots and five down-regulated protein spots was found as indicated by the arrowed spots in A of Fig. 2 and by the expanded plots in B of Fig. 2. Table S1 showed the average intensity values and their standard deviations of the spots and the fold differences between control and SB-treated group. The fold difference was represented by the ratio of the intensity value of SB-treated group to the value of control group. MS/MS analysis results of the differentially expressed proteins were also summarized in Table S1. The protein score, coverage and best ion score of each protein were showed. The result of MALDI-TOF MS/MS analysis of spot 4 was shown in Fig. 3 as an example.

### Confirmation of change in expression level and phosphorylation of proteins by Western blotting

**Figure 4:**
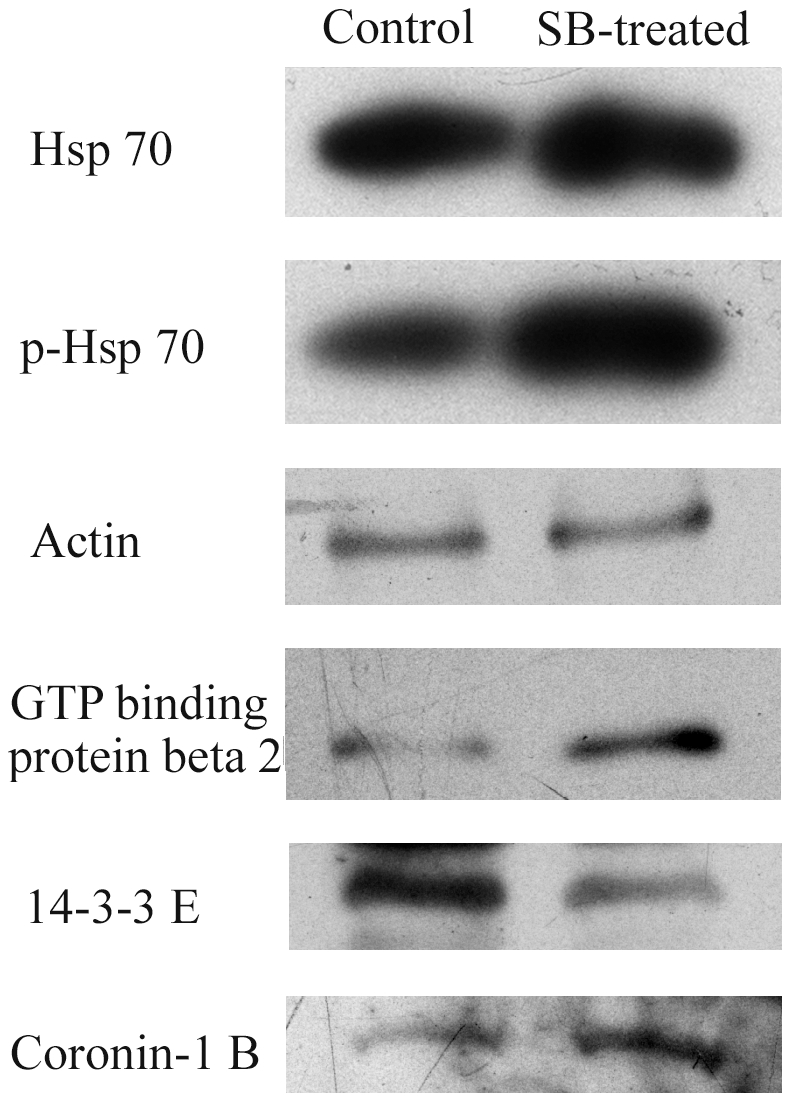
Results of Western blotting analysis. Protein levels of hsp 70, p-hsp70, guanine nucleotide binding protein beta, 14-3-3E and coronin-1B were checked. Each blot is the representative result of three independent experiments.

As shown in Fig. 4, hsp 70 protein was up-regulated in SB-treated platelets and, more importantly, phosphorylated hsp 70 level was also obviously up-regulated. Besides, 14-3-3E was found to be down-regulated while guanine nucleotide binding protein beta subunit and coronin-1B were found to be up-regulated in SB-treated platelets. These results were consistent with the proteomic result.

### Possible signal cascades network in platelets after binding of SB

**Figure 5:**
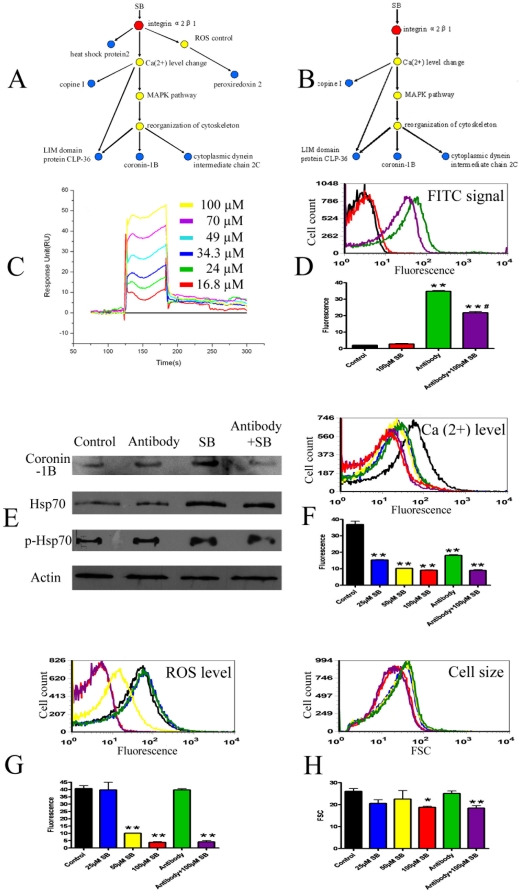
Prediction and certification of signal network of SB after binding with integrin α2β1. A. Predicted signal network of SB. The red dot illustrates integrin α2β1 while the blue dots are un-direct target-related proteins found in the proteomic study. The intermediate partners are shown in grey. B. Revised signal network of SB. C. Real time binding affinity measurement of SB to the integrin α2-I protein *in vitro* using Biacore 3000. Representative sensorgrams were obtained from injections of SB at concentrations of 16.8, 24, 34.3, 49, 70 and 100 µM (curves from *bottom* to *top*) over the immobilized integrin α2-I surface. D. Influence of SB treatment on binding between integrin α2β1 on platelets and its specific FITC-conjugated antibody. Both representative flow cytometry analysis result and quantitative results were shown. E. Western blotting analysis of protein levels of coronin-1 B, hsp70 and p-hsp70 in platelets under SB treatment with or without pretreatment of antibody against integrin α2β1. F. Flow cytometry analysis result of intracellular Ca(2+) level in platelets under SB treatment with or without pretreatment of antibody against integrin α2β1. Both respresentative flow cytometry analysis result and quantitative results were shown. G. Flow cytometry analysis result of intracellular ROS level in platelets under SB treatment with or without pretreatment of antibody against integrin α2β1. Both representative flow cytometry analysis result and quantitative results were shown. H. Flow cytometry analysis result of cell size of platelets under SB treatment with or without pretreatment of antibody against integrin α2β1. Both representative flow cytometry analysis result and quantitative results (FSC values) were shown. *Significant difference from the control group at P<0.05, **Significant difference from the control group at P<0.01, ^#^Significant difference from the antibody treatment group at P<0.05.

In our previous proteomic study, the effect of a mixture of salvianolic acids on the protein expression profile of platelets was checked and 12 differentially expressed proteins were found [11]. By comparing the 12 proteins found in the previous study with the 20 proteins found in the present study, we could identify 6 proteins that appeared in the both studies. They were heat shock-related 70 kDa protein 2 (heat shock protein 2), LIM domain protein CLP-36, copine I, peroxiredoxin-2, coronin-1 B and cytoplasmic dynein intermediate chain 2C. Interestingly, these 6 proteins were closely related to integrin α2β1, the possible direct target protein of SB identified in our previous study [9]. The possible signal cascades network in platelets after binding of SB to integrin α2β1 was predicted (A of Fig. 5). After binding to integrin α2β1, SB might induce regulation of its un-direct target-related protein through direct activation (heat shock protein 2), regulation of Ca(2+) level (copine I), ROS level (peroxiredoxin-2) and reorganization of cytoskeleton structure (LIM domain protein CLP-36, coronin-1 B and cytoplasmic dynein intermediate chain 2C).

### Certification of binding affinity of SB to integrin α2β1 *in vitro* and *ex vivo*

To verify the prediction that SB could bind directly to integrin α2β1 protein, the binding affinity of SB towards integrin α2β1 *in vitro* was determined by using Surface Plasmon Resonance (SPR) Biosensor analysis. The binding ability of SB towards integrin α2-I domain was reflected by response unit (RU) values recorded by the Biacore 3000 instrument. As shown in C of Fig. 5, RU increased with increasing SB concentration, which indicated that SB was able to bind to integrin α2-I in a dose-dependent manner. Binding of SB to integrin α2-I exhibited steady state. The equilibrium dissociation (*K*_D_) constant of SB binding to the immobilized integrin α2-I was 442×10^−6^ [M]. The curve fitting efficiency was evaluated by statistical parameter Chi^2^, a statistical parameter in SPR assay. The Chi^2^ value was calculated to be 2.28.

The binding affinity of SB to integrin α2β1 located on platelets was checked *ex vivo* in platelets. As shown in D of Fig. 5, a FITC-conjugated antibody against integrin α2β1 could bind to platelets and exhibited FITC fluorescence in flow cytometry analysis. Co-incubation of SB and the antibody with platelets could significantly decrease the amount of antibody binding on the platelets which was indicated by decrease in FITC fluorescence values of platelets. The results suggested that SB could compete with the antibody for the binding site of integrin α2β1 on platelets.

### Checking the effects of SB on platelets with or without pre-treatment of antibody against integrin α2β1

To confirm whether SB-caused changes in protein levels, intracellular Ca(2+) level, ROS level and cell size were dependent on its binding with integrin α2β1, the protein levels of coronin-1 B, hsp70, p-hsp70 and the intracellular Ca(2+) level, ROS level, cell size were checked in platelets with or without pre-treatment of antibody against integrin α2β1. As shown in E of Fig. 5, pre-treatment of antibody against integrin α2β1 blocked the increase of coronin-1 B but did not affect the increase of hsp70 as well as that of p-hsp70 caused by SB. The results indicated that the increase of coronin-1 B might be dependent on binding of SB with integrin α2β1. As shown in F of Fig. 5, the fact that antibody treatment alone could induce decrease of intracellular Ca(2+) level indicated that binding of integrin directly cause change in Ca(2+) equilibrium. In platelets treated with both antibody and SB, the decrease of intracellular Ca(2+) level might be result from combination of the effects of antibody and SB. On the contrary, antibody against integrin α2β1 showed no effect on ROS level and also no effect on SB-caused ROS level decrease (G of Fig. 5). The results indicated that the effect of SB on ROS might not be dependent on integrin α2β1. For the cell size, as shown in H of Fig. 5, though antibody alone did not induce significant change in cell size, combination of antibody and SB treatment enhanced the effect of SB on cell size.

According to above results, the predicted signal network was revised. The revised network of SB after binding with integrin α2β1 was shown in B of Fig. 5.

### Effect of SB on cytoskeleton structure of platelets

**Figure 6:**
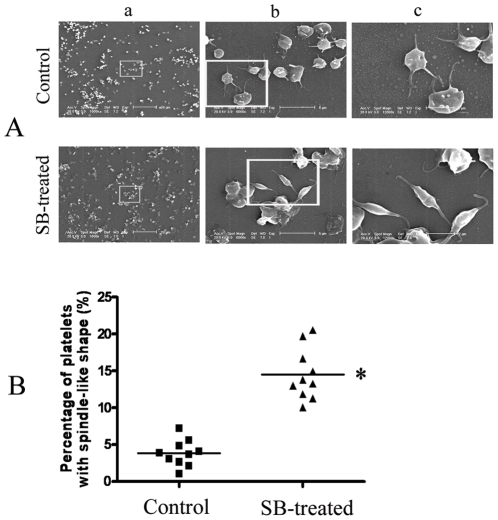
Effect of SB treatment on the shape of platelets. A, Representative result of scanning electron microscopy analysis. (a), Magnification 1000X, scale bar = 20 µm. (b), Higher magnification views of the boxed fields in (a), magnification 6000X, scale bar = 5 µm. (c) Higher magnification views of the boxed fields in (b), magnification 12000X, scale bar = 2 µm. B, The percentage of platelets with spindle-like shape in total platelets (result from 10 fields of view of 3 different experiments). *Significant difference from the control group at P<0.05.

Since the signal network suggested that SB might affect cytoskeleton structure and SB caused decrease in cell size as showed in flow cytometry analysis, further study using electron microscopic examination was conducted to check the effect of SB on platelets. As shown in A of Fig. 6, SB did induce change of cell shape in platelets. The control platelets was round or oval discoid while part of the platelets change to more slim shape like spindle-shaped after 100 µM SB treatment. Statistical analysis of the percentage of platelets with spindle-like shape in total platelets (result from 10 fields of view of 3 different experiments) indicated that the change in the shape of platelets after SB treatment was significant (B of Fig. 6).

## Discussion

In the present study, SB was found to inhibit the adhesion and aggregation of platelets induced by collagen. The effect of SB might be related to its binding to integrin α2β1, the possible direct target protein of SB found in our previous study [9]. The un-direct target-related proteins of SB in platelets were searched in the present study using a differential proteomic study. Proteomic technology has become an indispensable and efficient tool in biochemical research. And, this technology has successfully used to study signal pathways in platelets [12], [13]. Twenty possible target-related proteins of SB in platelets were found in the present study. Among the 20 proteins, 6 proteins including heat shock-related 70 kDa protein 2 (heat shock protein 2), LIM domain protein CLP-36, copine I, peroxiredoxin-2, coronin-1 B and cytoplasmic dynein intermediate chain 2C might be the most important ones and have close relationship with integrin α2β1 binding. The possible signal cascades network in platelets after binding of SB to integrin α2β1 was predicted based on previous reports and protein-protein interaction database. For example, previous works indicated that integrin α2β1 binding could cause change in calcium homeostasis and calcium-dependent functions [14], [15]. Previous report also suggested that, by binding to integrin α2β1, endorepellin could trigger transient activation of p38 mitogen-activated protein kinase (MAPK) and heat shock protein 27 and ultimately disassembly of actin stress fibers and focal adhesions [16].

To certify the predicted signal network, both the binding affinity of SB to integrin α2β1 and the influence of pretreatment with antibody against integrin α2β1 on the effects of SB in platelets were checked. The results indicated that SB had dose-dependently binding affinity to integrin α2β1 *in vitro* and could compete with antibody against integrin α2β1 for the binding site of integrin α2β1 on the membrane of platelets. Furthermore, flow cytometry analysis result confirmed that SB treatment caused dose-dependently decrease in intracellular level of Ca(2+) level and the effect of SB on Ca(2+) level might be related to integrin α2β1 binding. And, the change in coronin-1 B protein level caused by SB treatment was also dependent on integrin α2β1 binding. The revised signal network of SB including binding with integrin α2β1, change in Ca(2+) level, reorganization of cytoskeleton as well as change in cytoskeleton-related proteins such as coronin-1 B was established. As to the SB-caused change in ROS level and heat shock protein 70 level, our results indicated that these effects of SB might not be dependent on binding with integrin α2β1. For example, the decrease in ROS might be caused by the potency of SB to act as direct ROS scavenger. Based on its chemical structure characteristic, SB could exhibit direct antioxidative effect *in vitro* as well as *in vivo* [17], [18].

In the present study, SB was found to induce change in cytoskeleton of platelets. The flow cytometry analysis result showed that SB treatment caused decrease in platelet size and the scanning electron microscopic examination showed that SB treatment caused change in platelet shape. The change in cytoskeleton of platelets might be related to change in intracellular Ca(2+) level and subsequent regulation of proteins including copine I, coronin-1B, LIM domain protein CLP-36 and cytoplasmic dynein intermediate chain 2C. Copine I belongs to a ubiquitous family of calcium-dependent, membrane-binding proteins. In response to changes in intracellular Ca(2+), copines might regulate the activities and localization of their target proteins such as MEK1 [19], [20]. Previous reports suggested that integrin α2β1 binding caused reorganization of cytoskeleton in platelets [21], [22] through MAPK pathway [23], [24]. Coronins are a conserved family of WD repeat-containing, actin-binding proteins involved in modulating actin dynamics and cell motility [25], [26]. SB-induced increase in coronin-1 B in platelets was showed to be dependent on integrin α2β1 binding in the present study. LIM domain protein CLP-36 is a cytoskeletal and anchoring protein that has been suggested to be closely related to the plasma membrane Ca(2+)-ATPase, which plays an essential role in maintaining cytosolic Ca(2+) level in platelets [27]. Cytoplasmic dynein intermediate chain 2C is a component of the complex microtubule-based system of motor proteins in platelet [28].

In all, the present study identified 20 target-related proteins of SB in platelets through proteomic analysis. Signal network of SB from binding to its direct target integrin α2β1 was predicted and then certified. Binding of SB to integrin α2β1 caused change in level of intracellular Ca(2+), level of cytoskeleton-related proteins such as coronin-1 B and cytoskeleton structure of platelets. Signal cascades after SB binding to integrin α2β1 might be important basis for the inhibitive effect of SB on platelet adhesion and aggregation. To date, this study is the first to employ the proteomic technique to search globally for the proteins influenced in platelets by SB. The result of proteomic analysis and the suggested signal cascades network provided new hints for the study of the mechanism of SB on platelets.

## Materials and Methods

### Materials and reagents

SB was isolated and purified from the roots of *Salvia miltiorrhiza* by laboratory of phytochemistry, Shanghai Research Center for Modernization of Traditional Chinese Medicine, Shanghai Institute of Materia Medica, Chinese Academy of Sciences as reported in our previous paper [28]. SB was dissolved in natural saline for using in the following experiments. Natural saline was used in the following experiments as solvent control. All reagents used in two-dimensional electrophoresis were purchased from Bio-Rad Laboratories (Hercules, CA, USA). Other chemicals, except where specially noted, were purchased from Sigma-Aldrich Chemical Co. (St. Louis, MO, USA).

### Platelet preparation and adhesion assay

Platelet isolation was carried out as described previously [10]. Male SD rats (220–250 g) were bred and kept by the Laboratory Animal Services Center of Shanghai Institute of Materia Medica, Chinese Academy of Sciences. All procedures in animal experiments were conducted according to protocol approved by the Institutional Animal Care and Use Committee (IACUC) of Shanghai Institute of Materia Medica, Chinese Academy of Sciences. Platelet samples were permitted to be taken from anesthetized SD rats (male) for further study. The permit number for animal use in the present study is SIMM-AE-GDA-2010-02. Rats were anesthetized with sodium pentobarbital (50 mg/kg, i.p.). Blood samples were taken from the ventral aorta and transferred into plastic tubes containing 3.8% trisodium citrate as anticoagulant in a volume ratio 9:1. Platelet rich plasma was obtained by centrifugation of blood at 200 ×g for 10 min at room temperature. To avoid contamination with red blood cells and leukocytes, only the top one-third of platelet rich plasma was collected. Platelet-poor plasma was obtained by centrifugation at 1700 ×g for 10 min. Washed platelets were obtained by centrifugation of platelet rich plasma at 1000 ×g for 10 min and then wash the platelets twice with Tyrode buffer (136 mM NaCl, 2.7 mM KCl, 12 mM NaHCO3, 0.42 mM NaH_2_PO4, 0.2 mM MgSO_4_, 5.0 mM glucose) containing 0.2 mM EGTA, pH 6.5. The washed platelets were then centrifuged again at 1000 × g for 10 min and finally re-suspended in Tyrode buffer with a cell density of 3×10^8^ cells/ml. The contamination of red blood cells and leukocytes were measured to be <0.005% and <0.02% (in number), respectively.

Platelet adhesion assay was conducted similar to previous report [29]. Briefly, microplate wells were coated with 50 µg/ml collagen I (Sigma) overnight at 4 °C and washed twice with natural saline before use. After incubated with natural saline or SB at different concentrations at 37 °C for 5 min, 100 µl washed platelet suspension was plated into each microplate well and then incubated at 37 °C for 1 h in humidified thermostat. At the end of incubation, the microplate wells were washed twice with PBS and then rapidly supplemented with 150 µl of 0.1 M citrate buffer, pH 5.4, containing 5 mM p-nitrophenyl phosphate and 0.1% Triton X-100. After incubation at room temperature for 1 h, the reaction was stopped and the color was developed by the addition of 100 µl of 2 N NaOH. The p-nitrophenol produced by the reaction was measured with a microplate reader (Bio-Rad) at 405 nm against a platelet-free blank.

Platelet aggregation assay was performed using collagen as the aggregation inducer. Platelet rich plasma samples were obtained as described above and diluted with platelet poor plasma to a cell density of 3×10^8^ cells/ml. After incubated with natural saline or SB at different concentrations at 37 °C for 5 min, platelet rich plasma samples were put in silicone-treated glass cuvettes (200 µl/each cuvette). Then, aggregation of platelets was induced by addition of collagen (5 µg/ml) while stirring at 1000 rpm. The reaction was then allowed to proceed for 5 min, and the peak aggregation responses were measured using an aggregometer (Model TYXN-96, TongYong Corp., Shanghai, P.R.China).

### Proteomic analysis

#### Protein extraction

For protein sample preparation for proteomic analysis, washed platelets were incubated for 10 min with natural saline or 90 µM SB (dose of SB that is effective in inhibition of both platelet adhesion and aggregation). Subsequently, platelets were washed three times with PBS and then centrifuged for 10 min at 2500 × g. The pellets were dissolved in lysis buffer containing 7 M urea, 2 M thiourea, 2% CHAPS and 1% DTT, 0.8% Pharmalyte and protease-inhibitor (all from Bio-Rad). Homogenization of the cells was achieved by ultrasonication (10 strokes, low amplitude) on ice. The lysed cells were centrifuged at 15000 × g for 30 min at 4 °C and the supernatant containing the solubilized proteins was used for two-dimensional electrophoresis.

### Two-dimensional electrophoresis

Two-dimensional electrophoresis was carried out similar to our previous report [30] using Bio-Rad 2-DE system. Briefly, 150 µg protein sample was applied for IEF using the ReadyStrip IPG Strips, 17 cm, pH 4–7 (Bio-Rad). The strips were placed into a Protein IEF cell (Bio-Rad) and were rehydrated at 50 V for 12 h and then the proteins were separated based on their p*I*. After IEF, the IPG strips were equilibrated and then directly applied on to 12% homogeneous SDS-PAGE gels for electrophoresis using a PROTEIN II xi Cell system (Bio-Rad). The gels were then silver stained using Silver Stain Plus kit reagents (Bio-Rad).

### Differential image analysis

The silver-stained gels were scanned using a Densitometer GS-800 (Bio-Rad) and then analyzed using PD-Quest software (Bio-Rad). Comparisons were made between gel images of protein profiles obtained from SB-treated group and control group. The individual protein spot quantity was normalized as follows: the raw quantity of each spot in a member gel was divided by the total quantity of the valid spots in the gel, and normalized spot intensities were expressed in ppm. Quantitative analysis was performed using the Student's *t*-test between protein gels from control and SB-treated group. The significantly differentially expressed protein spots (*p*<0.05) with two fold or more increased or decreased intensity between control and SB-treated group were selected and subjected to further identification by MALDI-TOF MS/MS.

### In-gel digestion, peptide extraction and MALDI-TOF MS/MS

Proteins of interest were excised from the gels with EXQuest Spot Cutter (Bio-Rad) and placed into a 96-well microtitre plate. MS analysis was performed as reported before [30]. Briefly, gel pieces were de-stained, shrunk by dehydration in ACN and then digested with trypsin. Peptides were then extracted twice using 0.1% TFA in 50% ACN and the extracts were dried under the protection of N_2._ For MALDI-TOF MS/MS, peptides were mixed with 0.7 µl MALDI matrix and spotted on to the 192-well MALDI target plates. MS analysis was performed with an ABI 4700 Proteomics Analyzer MALDI-TOF/TOF mass spectrometer (Applera Applied Biosystems, Framingham, MA, USA). Mass spectra were obtained in a mass range of 700–3200 Da, using a laser (355 nm, 200 Hz) as desorption ionization source. After MS acquisition, the top five most intense peptides *per* spot were selected automatically for the MS/MS analysis, *i.e*., sequencing of these peptides. MS accuracy was externally calibrated with trypsin-digested peptides of horse myoglobin. The database search was performed by using the MASCOT search engine (Matrix Science, London, United Kingdom) to screen the NCBI protein sequence database restricted to rat taxonomy. Protein homology identifications of the top hit (first rank) with a relative score exceeding 95% probability and additional hits (second rank or more) with a relative score exceeding 98% probability threshold were retained. The probability-based score, assuming that the observed match is significant (*P*<0.05), had to be more than 50 when submitting PMF data to the database, and be more than 30 for individual peptide ions when submitting peptide sequence spectra.

### Western blotting analysis

Briefly, an aliquot (50 µg) of the extracted platelet protein samples was loaded onto a 12% SDS gel, separated electrophoretically, and transferred to a PVDF membrane (Bio-Rad). After blocking nonspecific protein binding with 10% dehydrated skim milk, the membrane was incubated with primary antibodies overnight at 4 °C. The primary antibodies used were mouse anti-Hsp70 (5A5) monoclonal antibody (1:200, Abcam, Cambridge, UK), rabbit anti-p-Hsp70 (Tyr 525) polyclonal antibody (1:200, Santa Cruz Biotechnology, California, CA, USA), goat anti-coronin 1B (C-19) polyclonal antibody (1:250, Santa Cruz Biotechnology, California, CA, USA), chicken anti-guanine nucleotide binding protein beta subunit polyclonal antibody (1:300, United States Biological, Massachusettes, MA, USA), rabbit anti-14-3-3E polyclonal antibody (1:500, Abgent, San Diego, CA, USA) and mouse anti-actin monoclonal antibody (1:2000, Sigma). Blots were then incubated with HRP-conjugated rabbit anti-goat IgG (1:2000, Immunology Consultants Laboratory, Newberg, OR, USA), HRP-conjugated goat anti-chicken IgY (1:1000, Immunology Consultants Laboratory, Newberg, OR, USA), HRP-conjugated goat anti-rabbit IgG (1:2000, Sigma) or HRP-conjugated goat anti-mouse IgG (1:2000, Sigma) for 1 h at room temperature and then visualized using chemiluminescence (Pierce Biotechnology, Rockford, IL, USA).

### Confirmation of binding between SB and EGFR *in vitro* with Surface Plasmon Resonance (SPR) Biosensor analysis

The binding affinity of SB to integrin α2β1 *in vitro* was assayed by the Drug Discovery and Design Center, Shanghai Institute of Materia Medica, Chinese Academy of Sciences using a SPR-based Biacore 3000 instrument (Biacore AB, Rapsgatan 7, S-754 50 Uppsala, Sweden) as reported before [30], [31]. The recombinant rat integrin α2 I domain (residues Pro138–Gly334) was expressed as a His- (C-terminal) and Strep-fusion (N-terminal) protein using *Escherichia coli*, and purified by affinity chromatography. The α2-I domain gene incorporating *Kpn*I and *Sac*I sites was synthesized by Shanghai Difa-Bio Ltd. (Shanghai, China). The *Kpn*I-*Sac*I fragment of the α2-I domain gene was subcloned into pET-51b expression vector (Novagen) and transformed into *E. coli* BL21(DE3) strain (Novagen). The BL21 transformants were cultivated in LB medium with ampicillin to an A_600_ nm of 0.8 at 37 °C, induced by adding isopropyl b-D-thiogalactopyranoside (IPTG) to a final concentration of 0.005 mM, and incubated overnight at 20 °C. The fusion protein, containing His-tag and Strep-tag, was isolated from bacterial lysates by Ni^2+^-chelation affinity chromatography using a HiTrape^TM^ Chelating HP column (GE Biosciences). The isolated protein was further purified using Introductory Strep-Tag II Kit (Novagen). This step improved purity of fusion protein to more than 95% (SDS-page analysis), and ensured the integrity of polypeptide. The purified recombinant a2-I domain was dialyzed against PBS buffer (KCl 2.7 mM, KH_2_PO_4_ 1.5 mM, NaCl 136.9 mM, Na_2_HPO_4_ 8.1 mM). Finally, the dialyzed protein solution was concentrated to more than 1 mg/ml for SPR analysis. In SPR analysis, the protein was dissolved in coupling buffer (10 mM sodium acetate, pH 3.5) and immobilized onto a CM5 sensor chip as ligand in 6171 RU with N-ethyl-N′-(3-dimethylaminopropyl) carbodiimide (EDC) and N-hydroxysuccinimide (NHS) according to the standard primary amine-coupling procedures. Biacore data were collected at 25 °C with HBS-EP buffer (10 mM HEPES, 150 mM NaCl, 3 mM EDTA, 0.005% (v/v) surfactant P20, pH 7.4) as the running buffer at a constant flow of 20 µl/min. SB was serially diluted into the running buffer with final concentrations of 16.8, 24, 34.3, 49, 70 and 100 µM. The SB samples were injected into the channels at a flow rate of 20 µl/min with injection time of 1 min, dissociation time of 2 min and followed by washing with the running buffer for 2 min at 30 µl/min. The binding responses were recorded continuously in response units (RU). The equilibrium dissociation constant (*K*_D_) was determined by analysis of the sensorgram-curves obtained at different concentrations of SB by use of BIA evaluation software version 3.1 (Biacore) and the 1:1 Langmuir binding fitting model. The curve fitting efficiency was evaluated by statistical parameter *Chi* ^2^.

### Certification of binding affinity of SB to integrin α2β1 *ex vivo* in platelets

The binding affinity of SB towards integrin α2β1 on platelets was determined by checking the influence of SB on binding between integrin α2β1 and its specific FITC-conjugated antibody. Washed platelets were suspended in Ca2+ and Mg2+ free Dulbecoo's phosphate-buffered saline and then treated with 100 µM SB, FITC-conjugated mouse anti-CD49b monoclonal antibody (1:200, BD Pharmingen) or cotreatment of antibody and 100 µM SB for 10 min. After washes with PBS, FITC fluorescence was measured using the Becton Dickinson FACScan flow cytometer (Becton Dickinson, La Jolla, CA, USA). The mean fluorescence intensity of at least 10, 000 of platelet was analyzed by Cellquest software (version 3.2).

### Checking the effects of SB on platelets with or without pre-treatment of antibody against integrin α2β1

Washed platelets were suspended in Ca^2+^ and Mg^2+^ free Dulbecco's phosphate-buffered-saline and then incubated for 10 min with 25, 50, 100 µM SB or 100 µM SB with pre-treatment of FITC-conjugated mouse anti-CD49b monoclonal antibody (1:200, 10 min). The protein levels of coronin-1 B, hsp70 and p-hsp70 in platelets were checked using Western blotting as described above. The intracellular Ca(2+) level as well as ROS level, cell size were checked using flow cytometry assay. Briefly, after twice PBS washes, platelets were incubated with 5 µM Fluo-3-AM (for measurement of the Ca(2+)) or 100 µM 2′7′-dichlorodihydrofluorescein diacetate (for measurement of the ROS level) for 10 min. After washes with PBS, fluorescence was measured using the Becton Dickinson FACScan flow cytometer (Becton Dickinson, La Jolla, CA, USA). The FSC (forward light scatter), correlating with cell size, was also checked in platelets. Data were collected and the mean fluorescence intensity of at least 10, 000 of platelet was analyzed by Cellquest software (version 3.2).

### Scanning electron microscopic examination

The scanning electron microscopic examination was conducted similar to previous report [32]. Briefly, after treated with 100 µM SB or natural saline for 10 min, platelets were fixed with 2% glutaraldehyde for 30 min. After twice washes in distilled water, the fixed platelets were layered on poly-L-lysine (1 mg/ml, MW 60,000) coated cover slips and allowed to settle in a humidified chamber for 12 h at 4 °C. Then, the specimens were post-fixed in 1.0% osmium tetroxide in phosphate buffer (pH 7.4) for 2 h, dehydrated with graded ethanol solutions and then evaporated in a critical point apparatus. The specimens were then mounted on aluminum copper stubs, and coated with 20 nm gold in a sputter coater (HCP-2 model, Hitachi, Tokyo, Japan). Observations were made with an environmental scanning electron microscope (XL-30 model, Philip, Eindhoven, Netherlands).

### Statistical analysis

For proteomic analysis, paired (control and SB-treated) protein samples from 3 independent experiments were analyzed. And, for each pair of protein samples, triplicate electrophoreses were performed to ensure reproducibility. For other analysis, results from at least 3 independent experiments were used for statistical analysis. Data are expressed as mean ± SD. Statistical significance was evaluated with the non-paired Student's *t*-test for comparison between 2 means. Probability values <0.05 were considered as significantly different.

## Supporting Information

Table S1 (0.06 MB DOC)
