# Supplementary material for: Differential Proteomic Analysis of Platelets Suggested Possible Signal Cascades Network in Platelets Treated with Salvianolic Acid B
Source: PLoS One. 2011 Feb 17;6(2):e14692. doi: 10.1371/journal.pone.0014692 (PMC3040754; doi:10.1371/journal.pone.0014692)
Supplement: Table S1 — (0.06 MB DOC) [file pone.0014692.s001.doc]

**Table 1.** List of possible target-related proteins of SB in platelets.

| Spot | Spot volume (ppm) | | Fold change | Target protein | Accession no. of NCBI database | Theoretical molecular mas*s* (kDa)/ p*I* | Protein score | Sequence coverage (%) | Best ion score | |
| --- | --- | --- | --- | --- | --- | --- | --- | --- | --- | --- |
|  | Control (Mean ± SD) | SB-treated (Mean ± SD) |  |  |  |  |  |  |  | |
| 1 | 354.2 ± 64.2 | 816.7 ± 210.4 | 2.31 | potassium voltage-gated channel, subfamily V, member 2 | 27684873 | 64.1/6.08 | 57 | 14% | | 32 |
| 2 | 172.3 ± 130.4 | 576.3 ± 246.4 | 3.34 | UMP-CMP kinase | 150383503 | 22.2/5.68 | 126 | 41% | | 34 |
| 3 | 76.0 ± 51.0 | 240.5 ±114.9 | 3.16 | ATP synthase alpha chain, mitochondrial precursor | 203055 | 58.8/9.22 | 103 | 42% | | 46 |
| 4 | 4608.5 ± 1503.2 | 2271.0 ± 629.6 | 0.49 | tyrosin 3-monooxygenase/tryptophan 5-monooxygenase activation protein (14-3-3 E) | 13928824 | 29.1/4.55 | 354 | 55% | | 94 |
| 5 | 365.9 ± 117.5 | 124.9 ± 71.9 | 0.34 | aldolase A | 6978487 | 39.3/8.31 | 80 | 35% | | 37 |
| 6 | 383.9 ±194.2 | 1096.7 ± 390.7 | 2.86 | LIM domain protein CLP-36 | 47940150 | 35.6/6.79 | 77 | 11% | | 33 |
| 7 | 191.6 ± 136.3 | 618.6 ± 238.8 | 3.23 | electron transfer flavoprotein subunit alpha, mitochondrial | 57527204 | 34.9/8.62 | 136 | 54% | | 67 |
| 8 | 382.4 ± 157.7 | 935.6 ± 256.4 | 2.45 | guanine nucleotide binding protein beta 2 | 71089941 | 35.0/5.91 | 58 | 17% | | 43 |
| 9 | 118.6 ± 91.6 | 359.0 ± 125.7 | 3.03 | tropomyosin 5 | 9653293 | 28.9/4.72 | 92 | 33% | | 84 |
| 10 | 1182.0 ± 215.3 | 474.6 ± 123.9 | 0.40 | zinc finger protein 29 homolog | 27710302 | 22.6/9.37 | 54 | 27% | | 33 |
| 11 | 951.9 ± 379.3 | 346.1 ± 200.1 | 0.36 | S-adenosylhomocysteine hydrolase | 8392878 | 47.5/6.07 | 130 | 22% | | 49 |
| 12 | 454.3 ± 166.4 | 1517.4 ± 727.1 | 3.34 | eukaryotic translation elongation factor 1 gamma | 62641860 | 72.4/8.64 | 176 | 44% | | 37 |
| 13 | 129.1 ± 24.2 | 390.0 ± 71.8 | 3.02 | copine I | 62646212 | 65.3/5.58 | 111 | 9% | | 58 |
| 14 | 544.6 ± 143.4 | 1269.1 ± 474.5 | 2.33 | heat shock-related 70 kDa protein 2 | 51858601 | 69.6/5.51 | 135 | 18% | | 31 |
| 15 | 245.0 ± 189.4 | 894.0 ± 181.8 | 3.65 | coronin-1B | 9506507 | 53.8/5.65 | 111 | 20% | | 37 |
| 16 | 270.9 ± 135.7 | 795.7 ± 242.6 | 2.94 | heparan sulfate (glucosamine) 3-O-sulfotransferase 3A1 | 62656233 | 43.4/10.11 | 107 | 31% | | 31 |
| 17 | 61.1 ± 80.5 | 241.1 ± 44.9 | 3.95 | cytoplasmic dynein intermediate chain 2C | 1151095 | 68.2/5.17 | 143 | 19% | | 54 |
| 18 | 289.8 ± 217.9 | 656.9 ± 260.8 | 2.27 | platelet-activating factor acetylhydrolase IB subunit beta | 11693154 | 25.6/5.57 | 60 | 12% | | 33 |
| 19 | 53.3 ±15.3 | 148.4 ± 43.7 | 2.78 | prothrombinase FGL2 | 11991263 | 40.9/7.1 | 70 | 10% | | 39 |
| 20 | 115.3 ± 51.3 | 41.4 ± 27.9 | 0.36 | peroxiredoxin 2 | 34849738 | 21.8/5.34 | 93 | 39% | | 57 |
